# The Effect of Carbon-Based Nanofillers on Cryogenic Temperature Mechanical Properties of CFRPs

**DOI:** 10.3390/polym16050638

**Published:** 2024-02-27

**Authors:** Aldobenedetto Zotti, Simona Zuppolini, Anna Borriello, Valeria Vinti, Luigi Trinchillo, Mauro Zarrelli

**Affiliations:** 1 Institute for Polymers, Composites and Biomaterials, National Research Council of Italy, P.le Fermi, 1, 80055 Portici, NA, Italy; aldobenedetto.zotti@unina.it (A.Z.); simona.zuppolini@cnr.it (S.Z.); mauro.zarrelli@cnr.it (M.Z.); 2 Avio S.p.A., Via Leonida Bissolati, 76, 00187 Roma, RM, Italy; valeria.vinti@avio.com (V.V.); luigi.trinchillo@avio.com (L.T.)

**Keywords:** nanocomposites, carbon-based nanofillers, mechanical properties, cryogenic temperature properties

## Abstract

In the present work, the effects of carbon-based nanofillers (0.5 wt%), i.e., graphene nanoplatelets (GNPs), carbon nanofibers (CNFs), and carbon nanotubes (CNTs), on the cryogenic temperature (77 K) mechanical properties of carbon fiber reinforced polymers (CFRPs) were investigated. The study utilized an ex situ conditioning method for cryogenic tests. The nanofillers were mixed with the epoxy matrix by a solvent-free fluidized bed mixing technique (FBM), while unidirectional carbon fibers were impregnated with the resulting nanocomposites to manufacture CFRP samples. Optical microscopy was employed to analyze the dispersion of the carbon-based fillers within the matrix, revealing a homogeneous distribution in nanocomposites containing GNPs and CNFs. Fracture toughness tests confirmed the homogeneity of the GNP-loaded systems, showing an improvement in the stress intensity factor (KC) by 13.2% and 14.7% compared to the unmodified matrix at RT (25 °C) and 77 K, respectively; moreover, flexural tests demonstrated a general increase in flexural strength with the presence of carbon-based nanofillers at both temperature levels (RT and 77 K). Additionally, interlaminar shear strength (ILSS) tests were performed and analyzed using the same ex situ conditioning method.

## 1. Introduction

Carbon fiber reinforced polymer (CFRP) composites have gained widespread usage in various industrial sectors such as aerospace [1,2], automotive [3], and naval [4] due to their exceptional specific mechanical properties, including mechanical property to weight ratio [5], in addition to their chemical [6] and thermal stability [7,8] when compared to traditional materials like steel, iron, and aluminum alloys. As a result, extensive research has been conducted to understand the mechanisms occurring in standard and adverse environments, such as moisture, UV radiation, and extreme temperatures [9], which these materials may encounter in their service life. For example, CFRPs show an exceptional potential to replace steel in bridge cables, underground oil extraction, and ocean engineering [10]. In particular, there are extreme conditions that require CFRP composites to operate at extremely low temperatures, such as cryogenic temperatures, which can significantly impact their response and behavior compared to standard conditions. Applications of CFRP composites in cryogenic temperatures include liquid propellant tanks, satellite components, aircraft operating at high altitudes, and insulating materials for superconducting magnets and structures for Arctic exploration [11]. Although there is no universally accepted definition for the temperature at which cryogenic temperatures end and higher temperatures begin, the threshold of −150 °C is commonly used in the literature as it is below which many commonly used gases, such as nitrogen, oxygen, hydrogen, and helium, are in a liquid state. Therefore, the temperature range between −273 °C (0 K) and −150 °C (123 K) is considered the cryogenic temperature range, while temperatures ranging from −150 °C to 50 °C (223 K) are classified as low temperatures (LTs) [12].

Due to the disparity in the coefficient of thermal expansion (CTE) between the fiber and matrix in composites, thermal stresses are generated, which can lead to the formation of microcracks and debonding at the fiber/matrix interface [13]. As a result, considerable attention has been paid to composite materials known as carbon fiber reinforced polymers (CFRPs) to investigate their mechanical properties and failure modes under cryogenic temperatures (CTs). The elastic modulus and tensile strength of the polymer matrix tend to increase as the temperature decreases. This occurs because the reduction in temperature also reduces the chains’ mobility, which results in increased binding forces between macromolecules, thereby increasing polymer strength. Additionally, according to the time–temperature superposition principle, the longer the chains take to relax at lower temperatures and at CTs, the more it is possible to consider the relaxation completely arrested, leading to increased stiffness [14].

At CTs, the polymer matrix loses its ductility and increases its stiffness, which results in a decrease in elongation at break and fracture toughness [15]. The work of Zhao et al. [16] supports these statements, as they investigated the effect of reducing the temperature from room temperature (RT) to 77 K on both the Young’s modulus (+37%) and tensile strength (+19%) of a DGEBF epoxy resin. However, the failure strain undergoes a significant reduction (−35%) at 77 K. For carbon fibers, as their level of crystallinity and molecular orientation are almost optimal, the increase in elastic modulus due to CTs is negligible; at the same time, carbon fibers undergo significant surface cracking at CTs, which leads to a reduction in fiber strength [17].

In carbon fiber reinforced polymers (CFRPs), it is observed that the tensile properties, specifically the longitudinal Young’s modulus and tensile strength, increase with a decrease in temperature. This phenomenon occurs despite the fact that the carbon fibers undergo a reduction in modulus due to surface microcracking. However, the fiber/matrix interface strength increases significantly due to the coefficient of thermal expansion (CTE) mismatch between the fibers (low CTE) and the matrix (large CTE). This justifies the increase in tensile properties observed in CFRPs [11]. Research conducted by Shi et al. [18] on the effect of three different testing temperatures (−196 °C, 25 °C, and 80 °C) on the tensile strength and modulus of epoxy-based CFRPs revealed that both the Young’s modulus and tensile strength increased at cryogenic temperatures (CTs) compared to room temperature (RT) properties. Similar behavior was observed for the flexural strength, with an increase of approximately 33% at CTs, which can be attributed to the matrix dominating CFRP flexural properties, and its strength tends to increase at CTs.

It is widely acknowledged [19,20] that the mechanical properties of carbon fiber reinforced polymers (CFRPs) are highly dependent on both the mechanical properties of the matrix and fibers, as well as the properties of the fiber/matrix interface. To enhance the mechanical properties of CFRPs at cryogenic temperatures (CTs), a common approach involves modifying the matrix with suitable fillers, which can improve the properties of both the matrix and fiber/matrix interface. Among the fillers utilized, nanoclays, carbon nanotubes (CNTs), graphene, and its derivatives are noteworthy [19,21]. It is important to recognize that any modification in the mechanical properties of modified polymers at room temperature (RT) does not necessarily imply the same trend at lower temperatures [22]. For example, Chen et al. [23] investigated the influence of CTs (77 K) on the tensile properties of epoxy nanocomposites containing CNTs (0.2 wt%), observing that the addition of CNTs resulted in a slight reduction in tensile strength at RT (−0.4%), while the trend reversed at CTs, with an increase of approximately +25.5%. Graphene oxide (GO) is commonly used to enhance the tensile and fracture properties of CFRPs at CTs. In the work of Qu et al. [19], GO was directly mixed with the epoxy matrix and the resulting CFRPs were examined for their flexural properties and interlaminar shear strength (ILSS) at RT and CT. The results demonstrated an improvement in flexural strength, flexural modulus, and ILSS of 6.4%, 9.6%, and 17.6%, respectively, at RT and 4.9%, 7.1%, and 8.7%, respectively, at CTs compared to the unmodified matrix.

Researchers including Hung et al. [24] have developed GO-based CFRPs using two methods: incorporating GO directly into the epoxy matrix and electrodepositing GO onto carbon fiber reinforcements. Both techniques have resulted in improved mechanical properties, with electrodeposition yielding the best results, resulting in a 37.3% increase in tensile strength at room temperature and a 47.6% increase at cryogenic temperature due to stronger chemical bonds and physical interlocking between the carbon fibers and epoxy matrix. The addition of soft fillers such as thermoplastic and rubber particles has also shown promise in improving the CT mechanical properties of CFRPs. Studies by Nobelen et al. [25] have shown that both rubber and core–shell rubber particles can significantly reduce microcrack density in laminates exposed to cryogenic cycling. Furthermore, the study found that the content of soft fillers has a greater impact on the CT mechanical properties of CFRPs than the type of filler used (core/shell or rubber particles).

The aim of this research was to examine the influence of various carbon-based nanofillers on the low-temperature mechanical properties of CFRPs. The nanofillers chosen, namely GNPs, CNFs, and CNTs, were selected due to their exceptional thermal and thermo-mechanical properties, such as thermal conductivity and CTE, which promote dimensional stability at low temperatures and limit the formation of residual stresses. These fillers were combined with the matrix material through the solvent-free fluidized bed mixing technique (FBM), which allows for a homogeneous dispersion without the use of costly and environmentally harmful solvents during the manufacturing process. The impact of the carbon-based fillers on the fracture toughness properties of the matrix material was examined at both room temperature and 77 K. CFRP composites, utilizing carbon-based filler-modified resins as the matrix, were analyzed in terms of flexural properties and ILSS. The novelty of this manuscript is strictly related to the specific material, which was investigated at cryogenic temperatures and characterized by different typologies of nanofillers. Other works have reported cryogenic properties mainly for nanocomposites, and thus available databases related to the corresponding fiber-reinforced systems, which can be found in the literature, are poor or limitedly populated. In addition, the dispersion of nanofillers within the matrix, in this work, was performed by a solvent-free FBM technique, which reveals two interesting and powerful advantages, namely the attained final level of dispersion and its potential scalability for an out-of-lab production.

## 2. Materials and Methods

### 2.1. Materials

The epoxy resin system utilized in this work is HXE75 [26], a patented product. It possesses a room-temperature viscosity of approximately 10^4^ Pas and a minimum viscosity of around 1 Pas at approximately 100 °C. Three distinct carbon-based fillers, namely graphene nanoplatelets (GNPs), carbon nanofibers (CNFs), and carbon nanotubes (CNTs), were incorporated. Among these, the GNPs, which are commercially available under the name G4Nan, were obtained from Nanesa (Arezzo, Italy). According to the technical datasheet, they possess a flake thickness of roughly 8 nm and a specific surface area of approximately 56 m^2^/g. The CNFs, produced by Grupo Antolin (Burgos, Spain), have a fiber diameter of 20–80 nm and a specific surface area of 80–120 m^2^/g. The single-walled CNTs, NC7000, purchased from Nanocyl (Sambreville, Belgium), have a tube diameter and length of 9.5 nm and 1.5 µm, respectively, with an aspect ratio of approximately 1000 and surface area of about 250–300 m^2^/g, making it the filler with the highest surface area among the ones used. The carbon fibers utilized in the composite fabrication process are Toray T700 UD, which possess a tensile strength of 4900 MPa and a tensile modulus of 230 GPa.

### 2.2. Nanocomposite and Composite Manufacturing

FBM technique is commonly used for nanocomposite manufacturing [27]. This process implements high-frequency acoustic waves directly on filler powder to break down solid clusters, which are naturally present within the system. The FBM technique offers several advantages over traditional mixing techniques, such as being a dry process that does not require hazardous solvents, thus permitting the mixing of large quantities of materials at low cost and with a moderate temperature increase in the system preventing uneven hot spots. The treated powders were then mixed with the HXE75 epoxy matrix using an industrial high-shear mixer. The resulting uncured nanocomposites were divided into two batches. The first was poured into a stainless steel mold coated with a mold release agent and cured according to the matrix cure profile. The second batch was used to manufacture carbon fiber reinforced composites (CFRCs).

**Figure 1:**
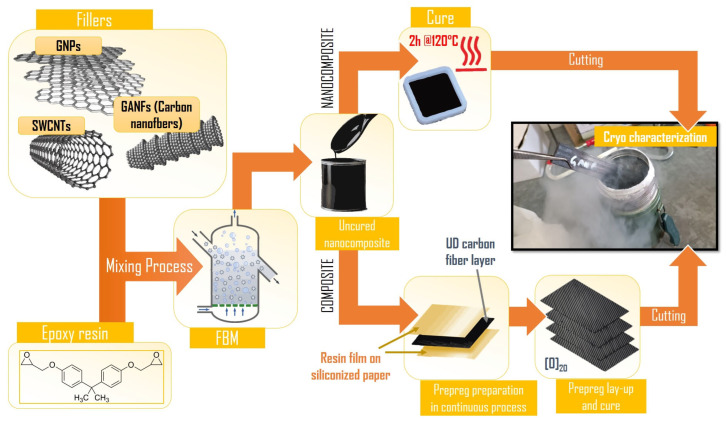
Manufacturing process of composites and nanocomposites for cryogenic characterization.

Composites prepregs were produced using a modified hot melting plant [27]. The prepreg manufacturing process was divided into two separate stages: (1) resin filming and (2) carbon fiber impregnation. The neat or modified resin was loaded in a filming apparatus, where it was deposited on siliconized paper by hot rollers. During the impregnation, the coated resin films passed through the impregnation machine sandwiching unidirectional carbon fiber reinforcement (T700 UD) on both sides. The process was conducted at optimized temperatures and speeds to ensure complete impregnation of the fibers with the polymer matrix. The manufactured prepregs were then rolled up and stored at −4 °C before being laminated according to the lay-up of the specific test standard. The CFRC manufacturing process is illustrated in Figure 1, and the complete list of realized nanocomposites and composites with corresponding labels is provided in Table 1.

### 2.3. Experimental Characterization

Optical micrographs of nanocomposites were acquired using an Olympus BX51 instrument (Tokyo, Japan) equipped with various magnification lenses. A high-speed saw was utilized to prepare 100 µm thick samples for optical microscopy examination.

**Figure 2:**
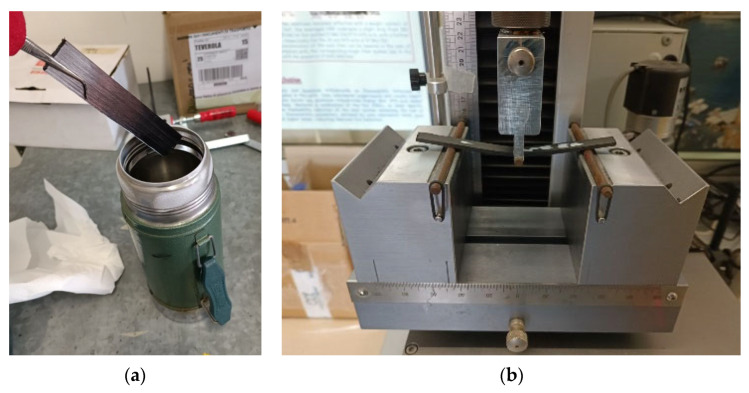
(**a**) Conditioning process in liquid nitrogen and (**b**) mechanical test (flexure) of the conditioned sample.

Mechanical tests were performed at both RT (25 °C) and cryogenic temperatures. Cryogenic temperature mechanical tests were performed using an ex situ conditioning method at −196 °C (77 K) [12,24,28]. To allow for property changes, samples were immersed in liquid nitrogen for 30 min (Figure 2a shows the immersion of the sample in the conditioning medium) prior to the mechanical test (Figure 2b). Afterward, the samples were transferred to the testing machine for immediate testing.

Mode-I fracture tests were conducted using the single-edge notched beam (SENB) geometry in accordance with the ASTM D5045 standard test method [29]. Samples with dimensions of 27 × 6 × 3 mm^3^ and a crack length (a) were selected such that 0.45 < a/W < 0.55, where W is the sample width, and were used to ensure plane strain conditions. The fracture tests were performed using an Instron 3360 dynamometer equipped with a 250 N load cell and a displacement rate of 10 mm/min. At least five specimens from each nanocomposite were tested at both RT and 77 K temperatures.

The ILSS values were evaluated according to the ASTM D2344 test standard [30] and calculated using the following equation:

(1) $$\mathrm{ILSS}\;(\mathrm{MPa})=\frac{3F}{4BW}$$

with F representing the maximum load (N) and B and W representing the thickness and width, respectively. Tests were conducted using a Lonos Tenso Test TT5 (Lonos Test, Monza, Italy) equipped with a 5 KN load cell and a constant crosshead speed of 1 mm/min. At least five specimens were tested for each temperature (RT and 77 K).

The ASTM D790 test standard [31] was employed for flexural tests conducted at both room temperature (RT) and 77 K, using a Lonos Tenso Test TT5 equipped with a 5 KN load cell. A span-to-depth ratio of 32:1 was used, and the crosshead speed was determined based on the equation provided in the test standard, which is:

(2) $$R=\frac{{ZL}^{2}}{2B}$$

where R is the crosshead speed (mm/min), Z is the rate of straining of the outer fibers (a constant value of 0.01), L is the support span, and B is the sample thickness.

## 3. Results

**Figure 3:**
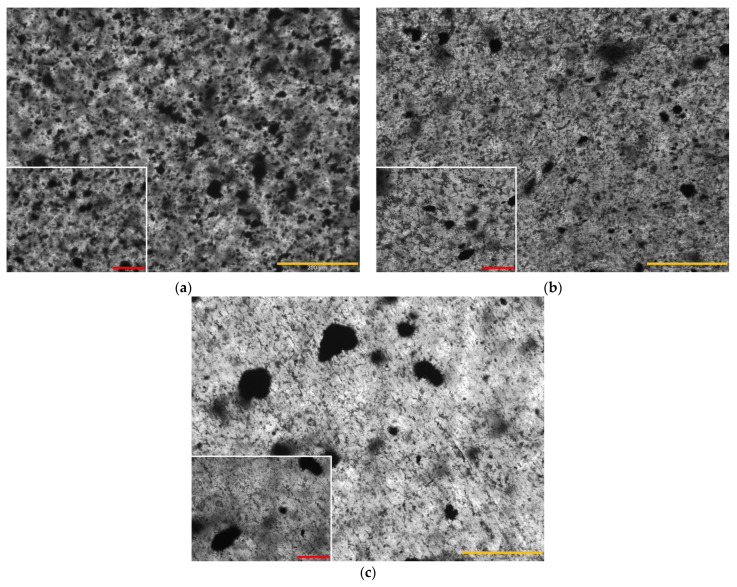
Optical microscopy of (**a**) HXE75 GNP, (**b**) HXE75 CNF, and (**c**) HXE75 CNT nanocomposites (yellow bar: 200 µm; red bar: 100 µm).

Carbon-based fillers tend to agglomerate [32], which limits their effectiveness as reinforcement in the polymer matrix. Therefore, in carbon-based nanocomposites, the dispersion process is critical, and the mixing temperature, time, and technique can significantly affect the filler’s reinforcing efficiency. Optical microscopy was used to assess the dispersion degree obtained using the fluidized bed mixing technique. Nanocomposites loaded with 0.5 wt% of GNPs showed good dispersion, with the largest clusters in the size range of 20–30 µm, as clearly shown in Figure 3a. The dispersion of CNFs in the epoxy matrix was slightly poorer than that obtained using GNPs, with the largest clusters being about 50–60 µm in size (Figure 3b), which was predictable considering that CNFs have a surface area almost double that of GNPs and tend to form clusters more easily. Given that CNTs have a surface area almost an order of magnitude larger than GNPs, it is easy to imagine that the CNT dispersion will be coarser than that obtained in GNP-based nanocomposites. Figure 3c shows large CNT clusters of about 100–150 µm in diameter, indicating that the fluidized bed mixing (FBM) method does not allow for good CNT dispersion.

**Figure 4:**
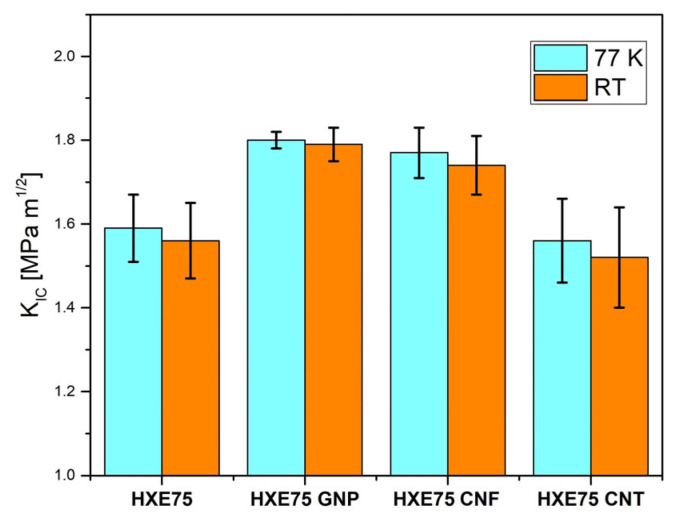
Critical stress intensity factor for the nanocomposites tested at 77 K and RT.

**Figure 5:**
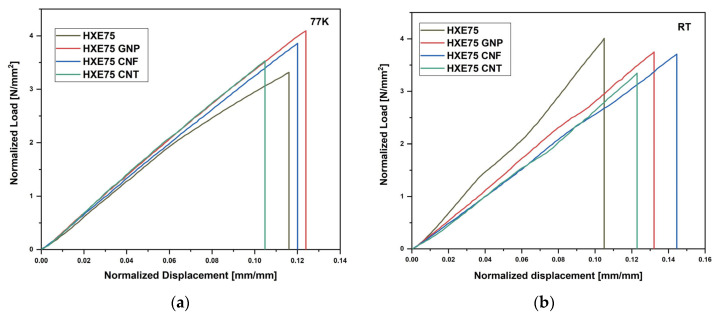
Fracture toughness raw data of tests performed at (**a**) 77 K and (**b**) RT.

The temperature-dependent critical stress intensity factor, K_IC_, for the examined nanocomposites is illustrated in Figure 4. The graph includes the average values and standard deviations calculated from five tests performed on each sample type. Normalized raw data of fracture toughness tests performed at different temperature are reported in Figure 5a (77 K) and Figure 5b (RT). Studies conducted by Nishijima et al. [33] have shown that the cryogenic temperature fracture toughness of an epoxy resin is higher than that evaluated at room temperature. This behavior is attributed to the existence of free space within the macromolecules despite the network shrinkage caused by the lower temperature, resulting in increased intermolecular forces between closer polymeric chains. A similar behavior was observed in our work, where fracture toughness values at 77 K were higher than those at room temperature, as depicted in Figure 3. Filler dispersion is a crucial parameter for matrix toughening, as both graphene nanoplatelets (GNPs) and graphene nanofibers (CNFs) increase fracture toughness at both 77 K and room temperature. This improvement is attributed to the homogeneous dispersion of carbon-based fillers in the epoxy matrix. In contrast, carbon nanotubes (CNTs) exhibit detrimental effects on system fracture toughness due to their inhomogeneous dispersion in the matrix. This results in a 1.89% and 4.66% reduction in K_IC_ at 77 K and room temperature, respectively. As shown in Table 2, the ΔK_IC_ values represent the percentage change relative to the unmodified matrix, indicating that no significant differences are observed at 77 K and room temperature. This suggests that the toughening efficiency for all investigated carbon-based fillers is not temperature-dependent in the analyzed temperature range.

**Figure 6:**
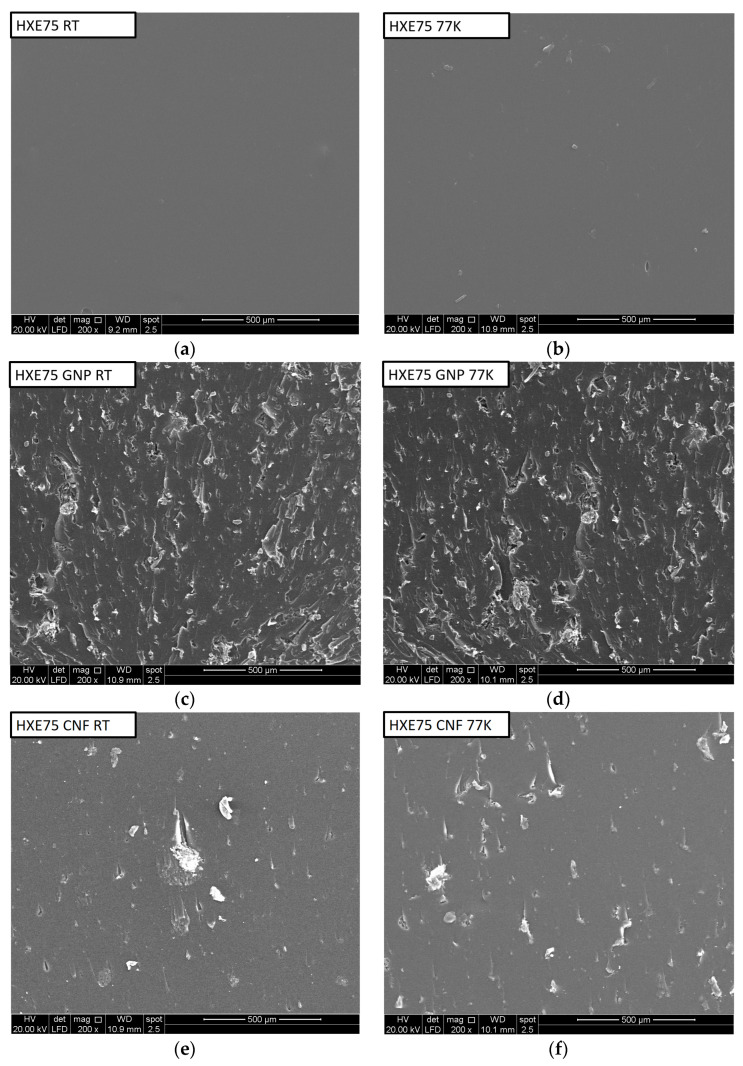

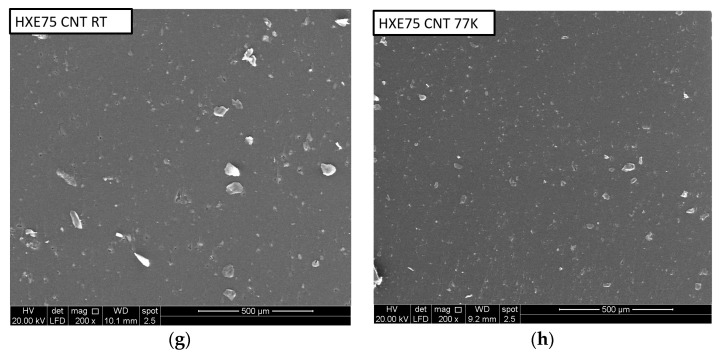
SEM images of nanocomposites fractured at 77 K and RT: (**a**,**b**) HXE75, (**c**,**d**) HXE75 GNP, (**e**,**f**) HXE75 CNF, and (**g**,**h**) HXE75 CNT.

The toughening mechanisms responsible for the increase in epoxy matrix fracture toughness by carbon-based fillers are crack pinning, bridging, and shear band formation [34], which are mechanisms that increase the energy dissipated through the increase in fracture surface roughness and plastic deformation. In order to investigate the related toughening mechanisms, fractured surfaces near the crack tip of the tested nanocomposites after fracture testing were examined by SEM (Figure 6). As shown in Figure 6a,b, the neat HXE75 matrix exhibits the typical smooth surface of brittle fracture at both 77 K and RT, indicating its poor fracture resistance. Concerning GNP- and CNF-loaded nanocomposites, a noticeably more tortuous surface is clearly observable in Figure 6c–f, which translates into the significantly improved fracture toughness as reported in Figure 4. Evidence of the crack pinning fracture mechanism is highlighted by the typical tails parallel to the crack propagation path: these tails are formed because fillers create obstruction to the propagation of the crack front and induce an increase in toughness by bowing out the crack front between the particles. In CNT-loaded nanocomposites, instead, the crack pinning tails are not observed because the CNT agglomerates are too large and weak, and clusters act as flaws rather than reinforcement (justifying the lowering of fracture toughness); moreover, in that case, the crack tends to propagate through particles, promoting a transparticle fracture mechanism. RT- and CT-fractured surfaces do not show any obvious difference in accordance with the fracture toughness values.

**Figure 7:**
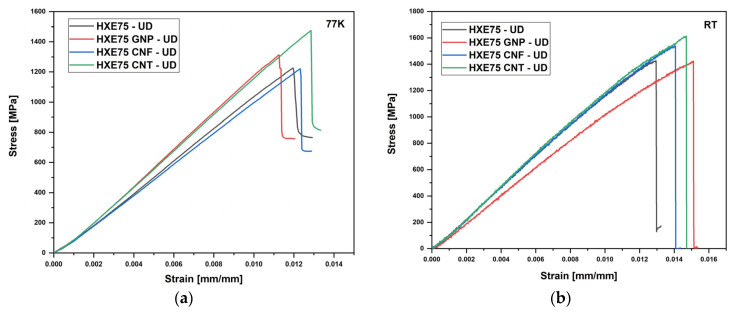
Flexural test raw data of tests performed at (**a**) 77 K and (**b**) RT.

**Figure 8:**
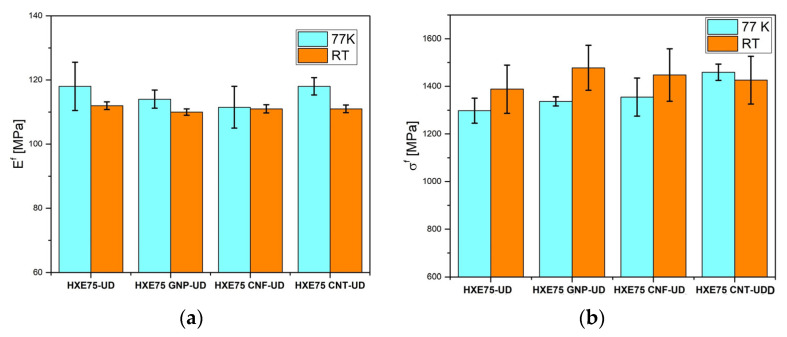
Flexural (**a**) modulus and (**b**) strength for the CFRCs tested at 77 K and RT.

The raw data shown in Figure 7 were employed to evaluate the flexural properties of the composites, and the graph shown in Figure 8 displays the flexural modulus and strength of four types of composites under examination at both room temperature (RT) and 77 K. The data for all specimens are also presented in Table 3. At RT, the addition of all fillers resulted in an increase in composite strength, with the greatest improvement observed in the GNP-loaded composite (+6.5); however, no significant changes in the flexural modulus were reported with the addition of carbon-based fillers. This behavior can be attributed to excessive reinforcement content, which leads to clustering at the fiber/matrix interface. The presence of these clusters at the interface can offset the general improvement in the modulus associated with the filler presence, limiting stress transfer between the fiber and matrix and, consequently, the increase in the flexural modulus. The clustering phenomenon was previously reported by Qu et al. [19], who observed an increase in flexural strength and modulus up to 0.2 wt% of filler content, followed by a strength reduction up to 0.5 wt%, at which the value is equal to that of the unmodified composite. At 77 K, the elastic modulus of the composites increased compared to the RT values due to the CTE mismatch between fibers and matrix, which enhances the stress transfer between the fiber and matrix. As shown in Figure 7b, the flexural strength undergoes a general reduction regardless of the presence of carbon-based fillers. This behavior can be explained by the fact that at low temperatures, local microcracks are difficult to extend due to the restriction of polymeric chains, leading to catastrophic failure without the occurrence of plastic deformation [13]. Additionally, despite the lower values at 77 K compared to RT, the flexural strength of carbon-based filler-loaded composites is higher than that of the unmodified ones. That behavior is comparable with the results reported by Hung et al. [24], who obtained an increase of 18% and 24%, at RT and 77 K, respectively, in flexural strength adding graphene oxide to the epoxy matrix of CFRPs. The flexural properties of laminates are strongly affected by the matrix properties, and it is especially noticeable by the analysis of the raw data in Figure 7; in fact, it is noteworthy that samples loaded at RT show a deviation by linearity at high strains due to the plastic deformation of the sample (Figure 7b). Conversely, the conditioned loaded samples show a linear behavior up to breakage, and this is associated with the lower mobility of polymeric chains at a cryogenic temperature, which hinders the plastic deformation of the matrix. That behavior was already reported by Qu et al. [19].

**Figure 9:**
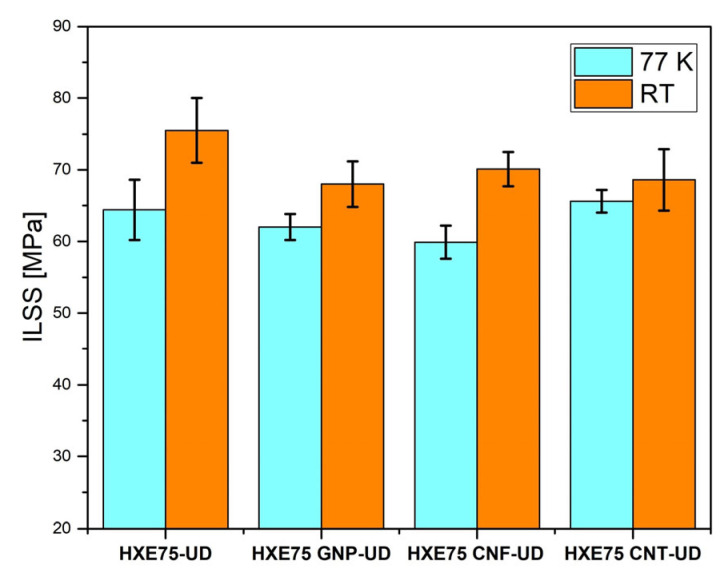
ILSS results for the nanocomposites tested at 77 K and RT.

Composite laminates underwent testing to evaluate their interlaminar shear strength (ILSS) properties, as indicated in Table 3 and Figure 9. The addition of carbon-based nanofillers resulted in a slight reduction in ILSS values compared to the neat matrix, which can be attributed to the dispersion of the nanofillers in the matrix, causing the formation of clusters that embrittle the interlaminar phase and promote transparticle fracture propagation [35].

The ILSS is extensively studied at cryogenic temperatures for commercial and industrial applications, as it is a fundamental parameter in the design of composite tanks for cryogenic transport [36]. However, there is no general trend for the variation in ILSS at low temperatures, as some studies report an increase in ILSS with a decreasing temperature [37], while others show the opposite trend [38]. This behavior is influenced by several factors, such as the fiber weight fraction [39] and the cryogenic characterization technique used. Many studies [40] report a reduction in cryogenic ILSS using ex situ conditioning, which is also observed in our work, where the ILSS reduction in the HXE75-UD sample is 14.7%. This reduction can be explained by the phenomenon of the interaction between the fiber and matrix and their different coefficients of thermal expansion (CTE). When the sample is suddenly exposed to room temperature (RT) after being held at 77 K, the fiber/matrix interface experiences a thermal shock, which leads to its embrittlement due to the generation of high residual stresses. These stresses induce the formation of cracks in the matrix and the fiber/matrix interface, promoting delamination during the ILSS tests. The presence of carbon-based fillers, at 77 K, does not induce significant variation compared to the RT trend.

Based on the experimental data analysis, it is clear that the major effect in terms of performance improvement is generated by the GNP filler due to the lower surface area and consequent more homogeneous dispersion, which play a critical role in microcrack formation at the fiber/matrix interface, determining a high enhancement of the RT and CT properties of the hosting system.

## 4. Conclusions

The influence of three different carbon-based nanofillers (GNPs, CNFs, and CNTs) on the mechanical properties of carbon fiber reinforced polymers at cryogenic temperatures (77 K) was examined through the use of an ex situ conditioning method for cryogenic temperature testing. Nanocomposites, realized by mixing the epoxy matrix (HXE75) and the fillers by the FBM techniques, were cured and characterized in terms of fracture toughness at both RT and CT. The results reveal the highest improvement for samples containing GNPs. However, the CNT-based nanocomposites showed a reduction in fracture toughness, primarily due to poor dispersion of the fillers in the matrix, resulting in large clusters (up to 100 μm in diameter). It was found that the fracture toughness values at 77 K were higher than those at RT, which can be attributed to the increased intermolecular forces between polymeric chains resulting from the thermal contraction of the polymer network.

Uncured resin was employed for the production of UD-CFRP laminates, which were characterized in term of flexural and interlaminar shear strength (ILSS) properties. The presence of carbon-based fillers resulted in a reduction in the flexural modulus of the carbon fiber reinforced polymer (CFRP), likely due to poor dispersion of the fillers, which hinders stress transfer between the fiber and matrix. At CTs, the increased stress transfer between the fiber and matrix (due to the CTE mismatch between the two phases) induces an increase in the flexural modulus (+5% for HXE75-UD). The results of the flexural tests show a general increase in flexural strength with the addition of carbon-based fillers, with the highest improvement observed for the GNP-loaded CFRP (+6.5% at RT). In general, the flexural strength values measured at 77 K were lower compared to those obtained at RT, which can be attributed to the formation of microcracks at the fiber–matrix interface due to thermal shock, promoting the reduction in both flexural strength and ILSS values. Based on the experimental results of the modified system, GNPs exhibited the best performance in terms of fracture toughness and flexural strength at both RT and 77 K, and this can be attributed to the better dispersion of GNPs in the hosting matrix.

## Figures and Tables

**Table 1:** List of manufactured samples.

Sample ID	Filler Typology	Filler Content [wt%]	Carbon Fiber Reinforcement
HXE75	-	0	-
HXE75 GNP	GNPs	0.5	-
HXE75 CNF	CNFs	0.5	-
HXE75 CNT	CNTs	0.1	-
HXE75-UD	-	0	T700 UD
HXE75 GNP-UD	GNPs	0.5	T700 UD
HXE75 CNF-UD	CNFs	0.5	T700 UD
HXE75 CNT-UD	CNTs	0.1	T700 UD

**Table 2:** Nanocomposite fracture toughness results obtained at 77 K and RT.

	77 K		RT	
	K_IC_ [MPa m^1/2^]	ΔK_IC_ [%]	K_IC_ [MPa m^1/2^]	ΔK_IC_ [%]
HXE75	1.59 ± 0.08	-	1.56 ± 0.09	-
HXE75 GNP	1.80 ± 0.02	13.2	1.79 ± 0.04	14.7
HXE75 CNF	1.77 ± 0.06	11.3	1.74 ± 0.06	11.5
HXE75 CNT	1.56 ± 0.10	−1.9	1.52 ± 0.11	−2.6

**Table 3:** CFRC flexural and ILSS test results obtained at 77 K and RT.

	E^f^ [MPa]		σ^f^ [MPa]		ILSS [MPa]	
	77 K	RT	77 K	RT	77 K	RT
HXE75-UD	118.3 ± 7.5	112 ± 1.2	1298 ± 53	1388 ± 101	64.4 ± 4.2	75.5 ± 4.5
HXE75 GNP-UD	114.1 ± 2.8	110 ± 1.0	1337 ± 19	1478 ± 95	62.0 ± 1.8	68.0 ± 3.2
HXE75 CNF-UD	111.5 ± 6.5	111 ± 1.3	1355 ± 80	1448 ± 110	59.9 ± 2.3	70.1 ± 2.4
HXE75 CNT-UD	118.2 ± 2.7	111 ± 1.2	1459 ± 34	1426 ± 100	65.6 ± 1.6	68.6 ± 4.3

## Data Availability

Data are contained within the article.

## References

[1] Soutis C. (2005). Carbon fiber reinforced plastics in aircraft construction. Mater. Sci. Eng. A.

[2] Elmahdy A., Zotti A., Zuppolini S., Zarrelli M., Borriello A., Verleysen V. (2021). Effect of Strain Rate and Silica Filler Content on the Compressive Behavior of RTM6 Epoxy-Based Nanocomposites. Polymers.

[3] Friedrich K., Almajid A.A. (2013). Manufacturing aspects of advanced polymer composites for automotive applications. Appl. Compos. Mater..

[4] Garcia-Espinel J.D., Castro-Fresno D., Gayo P.P., Ballester-Muñoz F. (2015). Effects of sea water environment on glass fiber reinforced plastic materials used for marine civil engineering constructions. Mater. Des..

[5] Qian D. (2010). Fiber-reinforced polymer composite materials with high specific strength and excellent solid particle erosion resistance. Wear.

[6] Ning Z., Liu R., El Hajjar R.F., Wang F. (2017). Micro-modeling of thermal properties in carbon fibers reinforced polymer composites with fiber breaks or delamination. Compos. Part B Eng..

[7] Liu L., Jia C., He J. (2015). Interfacial characterization, control and modification of carbon fiber reinforced polymer composites. Compos. Sci. Technol..

[8] Zotti A., Borriello A., Ricciardi M., Antonucci V., Giordano M., Zarrelli M. (2015). Effects of sepiolite clay on degradation and fire behaviour of a bisphenol A-based epoxy. Compos. Part B Eng..

[9] Kaw A.K. (2005). Mechanics of Composite Materials.

[10] Xian G., Guo R., Li C. (2022). Combined effects of sustained bending loading, water immersion and fiber hybrid mode on the mechanical properties of carbon/glass fiber reinforced polymer composite. Compos. Struct..

[11] Timmerman J.F., Tillman M.S., Hayes B.S., Seferis J.C. (2002). Matrix and fiber influences on the cryogenic microcracking of carbon fiber/epoxy composites. Compos. Part A Appl. Sci. Manuf..

[12] Sápi Z., Butler R. (2020). Properties of cryogenic and low temperature composite materials—A review. Cryogenics.

[13] Hung P.Y., Lau K.T., Fox B., Hameed N., Jia B., Lee J.H. (2019). Effect of graphene oxide concentration on the flexural properties of CFRP at low temperature. Carbon.

[14] Yang L., Li Z., Xu H., Wu Z. (2019). Prediction on Residual Stresses of Carbon/Epoxy Composite at Cryogenic Temperature. Polym. Compos..

[15] Ward I.M., Sweeney J. (2013). Mechanical Properties of Solid Polymers.

[16] Zhao Y., Chen Z.K., Liu Y., Xiao H.M., Feng Q.P., Fu S.Y. (2013). Simultaneously enhanced cryogenic tensile strength and fracture toughness of epoxy resins by carboxylic nitrile-butadiene nano-rubber. Compos. Part A Appl. Sci. Manuf..

[17] Zhang Y., Xu F., Zhang C., Wang J., Jia Z., Hui D. (2016). Tensile and interfacial properties of polyacrylonitrile-based carbon fiber after different cryogenic treated condition. Compos. Part B Eng..

[18] Shi H.Q., Sun B.G., Liu Q., Yang Z.Y., Zhang Y. Properties of cryogenic Epoxy Resin Matrix Composites prepared by RTM Process. Proceedings of the 20th International Conference on Composite Materials.

[19] Qu C.B., Huang Y., Li F. (2020). Enhanced cryogenic mechanical properties of carbon fiber reinforced epoxy composites by introducing graphene oxide. Compos. Commun..

[20] Zotti A., Zuppolini S., Borriello A., Trinchillo L., Vinti V., Zarrelli M. (2024). Hierarchical aerospace epoxy composites of carbon fiber and hyperbranched filler: Toughening behavior from nanocomposites to composites. Compos. Struct..

[21] Zotti A., Zuppolini S., Borriello A., Zarrelli M. (2022). Polymer nanocomposites based on Graphite Nanoplatelets and amphiphilic graphene platelets. Compos. Part B Eng..

[22] Chen D., Li J., Yuan Y. (2021). A Review of the Polymer for Cryogenic Application: Methods, Mechanisms and Perspectives. Polymers.

[23] Chen Z.K., Yang J.P., Ni Q.Q., Fu S.Y., Huang Y.G. (2009). Reinforcement of epoxy resins with multi-walled carbon nanotubes for enhancing cryogenic mechanical properties. Polymer.

[24] Hung P.Y., Lau K.T., Qiao K., Fox B., Hameed N. (2019). Property enhancement of CFRP composites with different graphene oxide employment methods at a cryogenic temperature. Compos. Part A.

[25] Nobelen M., Hayes B.S., Seferis J.C. (2003). Influence of elastomer distribution on the cryogenic microcracking of carbon fiber/epoxy composites. J. Appl. Polym. Sci..

[26] Di Cosmo A., D’andrea B., Vinti V. (2015). Polymeric Formulations with Chemically Adjustable Rheology for the Manufacture of Prepregs and Articles Made of Composite Material. U.S. Patent.

[27] Zotti A., Zuppolini S., Borriello A., Vinti V., Trinchillo L., Borrelli D., Caraviello A., Zarrelli M. (2022). Effect of the Mixing Technique of Graphene Nanoplatelets and Graphene Nanofibers on Fracture Toughness of Epoxy Based Nanocomposites and Composites. Polymers.

[28] Ma H.I., Jia Z., Lau K., Leng J., Hui D. (2016). Impact properties of glass fiber/epoxy composites at cryogenic environment. Compos. Part B.

[29] (2022). Standard Test Methods for Plane-Strain Fracture Toughness and Strain Energy Release Rate of Plastic Materials.

[30] (2022). Standard Test Method for Short-Beam Strength of Polymer Matrix Composite Materials and Their Laminates.

[31] (2017). Standard Test Methods for Flexural Properties of Unreinforced and Reinforced Plastics and Electrical Insulating Materials.

[32] Schulz S.C., Faiella G., Buschhorn S.T., Prado L.A.S.A., Giordano M., Schulte K., Bauhofer W. (2011). Combined electrical and rheological properties of shear induced multiwall carbon nanotube agglomerates in epoxy suspensions. Eur. Polym. J..

[33] Nishijima S., Honda Y., Okada T. (1995). Application of the positron annihilation method for evaluation of organic materials for cryogenic use. Cryogenics.

[34] Zotti A., Zuppolini S., Zarrelli M., Borriello A. (2016). Fracture toughening mechanisms in epoxy adhesives. Adhesives-Applications and Properties.

[35] Zotti A., Zuppolini S., Tábi T., Grasso M., Ren G., Borriello A., Zarrelli M. (2016). Effects of 1D and 2D nanofillers in basalt/poly (lactic acid) composites for additive manufacturing. Compos. Part B Eng..

[36] Islam M.S., Melendez-Soto E., Castellanos A.G., Prabhakar P. (2015). Investigation of Woven Composites as Potential Cryogenic Tank Materials. Cryogenics.

[37] Sethi S., Rathore D.K., Ray B.C. (2015). Effects of temperature and loading speed on interface-dominated strength in fibre/polymer composites: An evaluation for in-situ environment. Mater. Des..

[38] Takeda T., Shindo Y., Fukuzaki T., Narita F. (2014). Short beam interlaminar shear behaviour and electrical resistance-based damage self-sensing of woven carbon/epoxy composite laminates in a cryogenic environment. J. Compos. Mater..

[39] Kumar M.S., Sharma N., Ray B.C. (2009). Microstructural and Mechanical Aspects of Carbon/Epoxy Composites at Liquid Nitrogen Temperature. J. Reinf. Plast. Compos..

[40] Shukla M.J., Kumar D.S., Mahato K.K., Rathore D.K., Prusty R.K., Ray B.C. (2015). A comparative study of the mechanical performance of Glass and Glass/Carbon hybrid polymer composites at different temperature environments. IOP Conf. Ser. Mater. Sci. Eng..

